# An Overview of Thermal Infrared Imaging-Based Screenings during Pandemic Emergencies

**DOI:** 10.3390/ijerph18063286

**Published:** 2021-03-22

**Authors:** David Perpetuini, Chiara Filippini, Daniela Cardone, Arcangelo Merla

**Affiliations:** Department of Neuroscience and Imaging, Institute for Advanced Biomedical Technologies, University G. D’Annunzio of Chieti-Pescara, Via Luigi Polacchi 13, 66100 Chieti, Italy; david.perpetuini@unich.it (D.P.); chiara.filippini@unich.it (C.F.); arcangelo.merla@unich.it (A.M.)

**Keywords:** thermography, COVID-19, SARS-CoV-2, fever detection, pandemic, face recognition, machine learning, artificial intelligence

## Abstract

Infrared thermal imaging (IRI) is a contact-less technology able to monitor human skin temperature for biomedical applications and in real-life contexts. Its capacity to detect fever was exploited for mass screening during past epidemic emergencies as well as for the current COVID-19 pandemic. However, the only assessment of fever may not be selective for the Severe Acute Respiratory Syndrome Coronavirus 2 (SARS-CoV-2) infection. Hence, novel approaches for IRI data analysis have been investigated. The present review aims to describe how IRI have been employed during the last epidemics, highlighting the potentialities and the limitations of this technology to contain the contagions. Specifically, the methods employed for automatic face recognition and fever assessment and IRI’s performances in mass screening at airports and hospitals are reviewed. Moreover, an overview of novel machine learning methods for IRI data analysis, aimed to identify respiratory diseases, is provided. In addition, IRI-based smart technologies developed to support the healthcare during the COVID-19 pandemic are described. Finally, relevant guidelines to fully exploit IRI for COVID-19 identification are defined, to improve the effectiveness of IRI in the detection of the SARS-CoV-2 infection.

## 1. Introduction

The current outbreak of coronavirus disease 2019 (COVID-19), caused by the severe acute respiratory syndrome coronavirus 2 (SARS-CoV-2), has currently spread to over 200 countries, with around 120 million confirmed cases worldwide and more than 2.5 million deaths since the start of the outbreak (updated 11 March 2021) [1]. The coronavirus infection emerged in Wuhan, China, in December 2019. On 30 January 2020, the agency’s emergency committee by the World Health Organization (WHO) declared the outbreak as a public health emergency of international concern and on 11 March 2020, it was characterized as a pandemic, since the novel virus continued to rapidly spread worldwide [2].

Coronaviruses are enveloped, positive single-stranded large RNA viruses that infect a wide range of animals but also humans. They usually cause mild to moderate upper-respiratory tract illnesses, like the common cold. However, three new coronaviruses have emerged from animal reservoirs over the past two decades to cause serious and widespread illness eventually leading to death (i.e., SARS-CoV, MERS-CoV and SARS-CoV-2). 

SARS-CoV, emerged in late 2002, causing the severe acute respiratory syndrome (SARS), then spread rapidly around the world and infected around 8.098 people in 37 countries with 778 deaths. SARS-CoV disappeared by 2003 [3]. The symptoms of SARS-CoV were very severe, therefore it was quite simple to identify and isolate patients, quickly narrowing the epidemic spread. 

MERS-CoV, emerged in 2012, particularly in Saudi Arabia, the United Arab Emirates and the Republic of Korea and caused the Middle East Respiratory Syndrome (MERS). Transmission of MERS-CoV has mainly occurred in healthcare settings and continues to cause sporadic and localized outbreaks, especially in travelers from the Middle East. In total there were 2.519 cases and 858 deaths [4]. 

Unlike SARS-CoV and MERS-CoV, SARS-CoV-2 is much more widely transmitted in the community and has a higher pandemic potential [5]. SARS-CoV-2 infection is transmitted through large droplets generated by symptomatic patients during sneezing and coughing but can also occur from asymptomatic individuals and prior to symptoms onset [6]. Current estimates suggest a median incubation period of 5 to 6 days for COVID-19, with a variability range of 1 to 14 days [7]. Clinical presentation of the disease ranges from no symptoms (asymptomatic) to severe pneumonia which may lead to death. The most commonly reported clinical symptoms are fever (88%), followed by dry cough (68%), fatigue (38%), dyspnea (19%), sore throat (14%), headache (14%) and myalgia or arthralgia (15%). Less common symptoms include diarrhea (4%) and vomiting (5%). Approximately 80% of reported cases had mild to moderate symptoms, 13.8% had severe illness, and 6.1% had critical illness (respiratory failure, septic shock and/or multi-organ dysfunction/failure) [8]. In addition, there is presumably no pre-existing immunity to the new coronavirus in the population and everyone is expected to be susceptible.

Different strategies have been put in place to try to contain the spread of COVID-19 on a national and global scale, and several approaches for diagnosis and clinical treatments have been proposed [9,10]. Similarly to previous epidemics, infrared thermography (IRI) is currently employed to contain the outbreak of COVID-19, thanks to its contactless feature and to its capability to quickly assess skin temperature (T_sk_) variations and fever [11,12]. IRI is a non-invasive, contactless and low-cost technology that measures the radiation from a body, providing information on its superficial temperature. Importantly, IRI, measuring the infrared radiation emitted by every object with temperature above absolute zero, is a passive technology. Therefore, IRI does not require any dosage of radiation, making this technique completely safe. This technique found a wide field of application thanks to the technological improvement of the IR sensors [13]. In fact, IR devices are characterized by large focal plane array (FPA) detectors (up to 1280 × 1024 pixels), which guarantee to collect temperature maps (i.e., thermograms) with high Non-Equivalent Temperature Difference (NETD, ~30 mK) and dot pitch (~25–40 μm) [14]. In biomedical applications, IRI imaging allows to non-invasively record the human T_sk_, without physical constraints for the subjects. Particularly, this technique has been used in medicine to evaluate injuries [15,16,17], to support clinical diagnosis of different pathologies [18,19,20,21] and to detect the autonomic activity for psychophysiological assessments in healthcare and technological fields [22,23,24]. Concerning the IRI employment to limit the COVID-19 spread, since patients could be partially asymptomatic or do not exhibit fever as symptom, classical IRI evaluations are not always suitable to detect the infection. This review aims to provide an overview of the procedures and algorithms used for IRI-based fever detection, focusing on the points of strength and weakness of IRI application for mass screening during the last epidemics, and in particular during COVID-19 pandemic. Moreover, the present review reports about the novel approaches based on machine learning (ML) [25] which exploits both spatial and temporal features of IRI recordings to increase its capability to identify infected patients. Furthermore, novel IRI-based technologies aiming to limit the SARS-CoV-2 contagions are described. Finally, the IRI potentialities to provide a valid tool to support the early identification of the infection, even in asymptomatic cases, are discussed. 

## 2. Study Organization and Search Processing Method

The guidelines proposed by Kitchenham [26] have been followed for this narrative review, with the aim to investigate the employment of IRI during pandemics, focusing on the methods used for the mass fever screening. Moreover, an overview of the novel methodologies and technologies developed during the COVID-19 pandemic is provided. To this aim, the research questions (RQs) addressed by this overview are:

RQ1. What methods are employed for fever assessment? What factors could influence the IRI capability of fever detection? 

RQ2. What was the effectiveness of IRI fever mass screening in healthcare settings and airports in reducing the spread of the infections in previous epidemics?

RQ3. Could ML approaches deliver a more accurate detection of breathing apparatus infection even in asymptomatic cases? 

**Figure 1:**
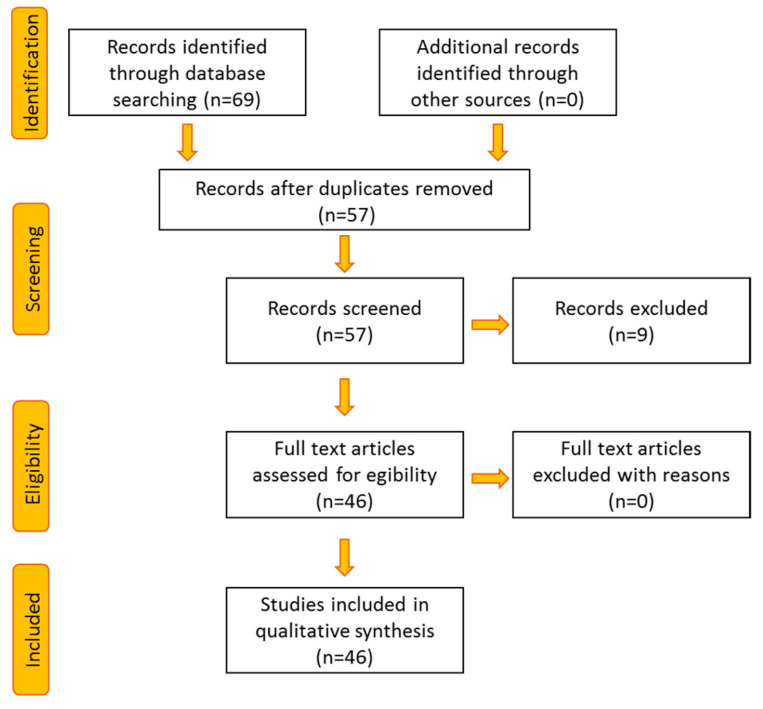
Literature screening procedure for the selection of papers included in the review.

The literature research was conducted on Scopus database, using the following keywords: “thermography” OR “thermal imaging” AND “Covid-19” OR “Sars-Cov-2” OR “Ebola” OR “pandemic” OR “fever screening”. The search was performed for the article title, abstract and keywords, and limited to the original articles (i.e., Conference paper, Review, Letter, Short Survey were excluded) concerning the subject areas Engineering, Computer Science and Medicine. The database provided 69 results, which were investigated through a manual review procedure to identify the papers suitable for this work. Particularly, papers not related to IRI data analysis, or automatic face recognition, or ROI selection for fever screening or disease classification purposes were excluded. After this review process, 46 papers were included in the study (Figure 1). The resulting papers were analyzed and grouped based on the operative RQs.

## 3. Methodologies for IRI-Based Fever Detection

### 3.1. External and Internal Confounding for Fever Assessment

IRI provides an indirect measure of the core temperature, relying on the relationship between T_sk_ and core temperature. The IRI performances in estimating the feverish condition are usually defined through several statistical metrics. Particularly, the sensitivity measures the amount of positives cases that are correctly identified, whereas the specificity is indicative of the proportion of the negative cases that are correctly classified. The positive predictive value (PPV) is the extent of positive results of a test that are true positive cases, whereas the negative predictive value (NPV) is the amount of negative results of a classification that are true negative cases. In order to reliably assess feverish conditions through IRI from T_sk_, some issues needed to be faced. 

Firstly, the capability to estimate fever from different facial regions have been investigated. Hausfater et al. [27] detected feverish state of the subjects, defined by tympanic temperature, through IRI from the forehead. The method delivered a sensitivity of 0.76, a specificity of 0.65, a positive predictive value (PPV) of 0.16, a negative predictive value (NPV) of 0.97, and an accuracy of 0.66. Zhou et al. [28] proposed a method for fever detection based on one RGB camera and two IRI systems, recording three consecutive frames with a sampling frequency of 30 Hz. The RGB camera allowed the face recognition and the definition of 17 regions of interest (ROIs) on the face. The maximum value of the ROIs was considered to classify the feverish status. The Receiver Operating Curve (ROC) analysis delivered an area under the curve (AUC) of 0.97, with a sensitivity of 0.85 and specificity of 0.89, considering oral thermometry as the gold standard. Moreover, Chen and colleagues [29] studied the capability to estimate the core temperature from different ROIs. Particularly, they employed a tympanic thermometer to measure the core temperature, and it was compared to the forehead and wrist temperatures. They demonstrated that wrist temperature cannot be used to establish a threshold for fever screening, whereas a fixed offset between tympanic and forehead temperature was found (~ 2 °C). Therefore, the authors proposed a standard operating procedure (SOP) for the measurement of body temperature using an infrared thermometer, suggesting a threshold for the forehead temperature of 36 °C for fever identification. Ring et al. [30] tested the IRI fever assessment capabilities evaluating the mean temperature of ROIs placed over the forehead and inner canthi. A good correlation between axilla and inner canthi of the eyes temperatures was found.

A second issue concerning the fever assessment through IRI is related to the confounding effects due to inaccuracies of the radiation model of the skin and to uncertainties of the measurement conditions. Hence, in order to perform a reliable and accurate fever assessment, external and internal confounding factors need to be considered. Dzien et al. [31] tested the capability of IRI to assess fever from forehead with different external environmental temperatures. The results indicated that the temperature measured through IRI devices from forehead is not an appropriate tool to detect infectious diseases directly at the entrance of a building, at least with cold outdoor temperatures. In order to minimize internal confounding effects on IRI measurements, Ghassemi et al. [32] developed a battery of evaluation test methods for standardized, objective and quantitative assessment of IRI performance, comparing two different thermal cameras. Moreover, they proposed optimized methods for estimating body temperature, demonstrating that IRI devices’ performance could be affected by many factors (e.g., location and size of the target plane and blackbody uncertainty) [33]. In order to limit the uncertainty of IRI measurements related to human skin radiation model, Chu et al. [34] proposed the joint maximum a posterior (JMAP) approach with a hierarchical prior model of the thermal radiation to be applied on human face, to provide an efficient and accurate evaluation of abnormal T_sk_. To limit the effects due to inhomogeneities of the measurement conditions, operational guidelines for identifying a febrile human using a screening thermograph (ISO/TR 13154:2009 ISO/TR 80600) have been defined. Ring and colleagues [35] used these guidelines to assess fever in children through IRI measurements from eyes inner canthi, forehead, and ear, comparing the performance with that of a clinical radiometer. A significant difference between the temperatures measured in non-fevered and fevered patients was assessed, and the thermal imaging of the eye region has been proved to be the most rapid contactless site for measurement.

### 3.2. Inner Canthi Identification and Face Segmentation Algorithms in Pandemic Outbreaks

Since the eyes’ inner canthi were demonstrated to be highly indicative of the core temperature researchers put the effort into defining automatic methods for inner canthi’s anatomical detection. Dwith et al. [36] developed a method based on coarse-fine registration strategy based on landmarks and edge detection on eye contours, employing co-registered infrared and RGB cameras. The registration accuracy was estimated within ±2.7 mm, which enables accurate localization of the canthi regions. Moreover, using co-registered IRI-RGB cameras, Dwith et al. [37] developed a method relying on free form deformation models based on the Demons and cubic B-spline algorithms. They found an error in the definition of the inner canthi of 2.8 ± 1.2 mm, and an error of the definition of the maximum temperature in the region of 0.10 ± 0.09 °C. Ferrari et al. [38] used a method based on sparse 2D-3D points correspondence using a 3D Morphable Face Model (3DMM) on thermal videos to define the inner canthi regions. Cardone et al. [39] proposed a method to automatically warp, through a Local Weighted Mean transformation, IRI images of faces over a template by means of simultaneous recordings of RGB and IRI cameras. Sixty-eight facial landmarks were identified in each frame in the visible domain through the open-source software OpenFace [40] and transformed into the corresponding frame of infrared images, allowing to identify anatomical landmarks over the IRI image of the faces with an RMSE of 0.66 pixels. Müller et al. [41] employed convolutional neural networks for multiclass segmentation in thermal infrared face analysis. The principle is based on existing image-to-image translation approaches, where each pixel in an image is assigned to a class label. The procedure allowed to correctly identify all the classes, including mask and glasses worn by the subjects. 

A further issue related to a fast, large-scale fever screening is associated to face recognition from a non-uniform background. Radzi et al. [42] proposed an algorithm based on Gaussian Bi-modal Mixture Models (GBMM) for background-foreground segmentation as an important feature to identify medial canthal area, to be exploited for fever mass screening.

Although an accurate estimation of the inner canthus position is provided, some external factors of the measurement conditions could affect the capability to properly collect their temperature. Vardasca et al. [43] identified the impact of using different distances and angles in the assessment of the average inner-canthi temperature. To minimize the measurement error, distances between 80 and 120 cm and angles as close as possible to 90° should be used when the inner-canthi of the eye temperature is recorded with conventional lenses and standard image analysis software for fever screening [43]. Vardasca et al. [44] also demonstrated, employing the Bland–Altmann limits of agreement, that the bilateral difference of the inner canthus of the eyes is negligible.

## 4. Mass IRI-Based Fever Screening in Public Environments

### 4.1. Mass Fever Screening in Hospitals

Thanks to IRI’s contactless features, its employment for fever mass screening is largely encouraged, particularly in healthcare and transport hubs, such as airports.

Regarding the employment of IRI systems in healthcare settings, Hewlett et al. [45] discriminated feverish subjects from healthy individuals with a sensitivity of 0.70, specificity of 0.92, PPV of 0.42, and NPV of 0.97. Moreover, Chiu et al. [46] investigated the effectiveness of the fever screening in hospital, obtaining a sensitivity of 75% and specificity of 99.6%. Bardou et al. [47] obtained sensitivity of 0.9286, specificity of 0.9967, PPV of 0.8667, and NPV of 0.9984 for feverish subjects detection. Nguyen et al. [48] compared the performances of three IRI systems for mass fever screening (i.e., FLIR ThermoVision A20M [FLIR Systems Inc., Boston, MA, USA], OptoTherm Thermoscreen [OptoTherm Thermal Imaging Systems and Infrared Cameras Inc., Sewickley, PA, USA], and Wahl Fever Alert Imager HSI2000S [Wahl Instruments Inc., Asheville, NC, USA]). Correlations of IRI temperatures and oral temperatures (expressed through the correlation coefficient, ρ), measured with DinaMap ProCare digital thermometer, were similar for OptoTherm (ρ = 0.43) and FLIR (ρ = 0.42) but significantly lower for Wahl (ρ = 0.14; *p* < 0.001). The AUC delivered by the ROC analysis for OptoTherm (0.96) and FLIR (0.92) were not significantly different but were significantly greater than the AUC of Wahl (0.78; *p* < 0.001). Chiang et al. [49] tested the fever assessment under different conditions, comparing an IRI device, a thermal scanner (i.e., thermoguard) and an IRI ear drum. A significant difference was found at 10 m distance between ear drum IRI and thermoguard, lateral view IRI, and frontal IRI. Through a ROC analysis, the optimal cut-off temperatures for the different imagers were defined as 36.05 °C for thermoguard (AUC of 0.716), 36.25 °C for lateral view IRI (AUC of 0.801), and 36.25 °C for frontal view IRI (AUC of 0.812).

Concerning COVID-19 pandemic, McConeghy et al. [50] developed a screening procedure based on the definition of a threshold of the skin temperature in two nursing homes. Employing a ROC analysis, they identified the best threshold as 38 °C, but they concluded that the only T_sk_ measurement is an insufficient tool to identify COVID-19 infection.

### 4.2. Mass Fever Screening in Airports

Concerning the large-scale screening in airports, Nishiura and Kamiya [51] described the performances of fever screening at Narita International Airport (Japan) during the the spread of the swine flu pandemic caused by the influenza A subtybe H1N1 virus. A logistic regression combined with a ROC analysis delivered sensitivity and specificity of the infrared thermoscanners in detecting hyperthermia ranged from 50.8–70.4% and 63.6–81.7%, respectively. Kuan et al. [52] studied the performances of the infrared thermometers to identify dengue fever patients in airport passengers in Taiwan during the period 1998–2007. This surveillance system successfully identified 45% of the cases; PPV varied from 30.5% to 62.6%. Kuan and Chang [53] demonstrated that 44.9% (95%CI: 35.73–54.13%) of the confirmed imported dengue cases with an apparent symptom (febrile) in the viremic stage were detected employing a IRI device during the period 2007–2010. The estimated PPV was of 2.36% (95% CI: 0.96–3.75%) and the NPV was > 99.99%. Shu et al. [54] demonstrated that IRI devices were able to identify 65.8% of dengue cases in airport screening. Cho and Yoon [55] tested the reliability for fever screening of IRI comparing its performance with tympanic measurements. No statistical differences between tympanic and IRI were found. During the SARS diffusion, Sun et al. [56] already developed a portable screening system designed for onboard entry screening at international airports. Face detection was performed automatically employing OpenCV libraries [57], and whole face single thermograms were used to classify the pathology. Using a Linear Discriminant analysis, sensitivity and NPV of 100% were obtained. The specificity and PPV were 88% and 33% respectively.

In order to provide an accurate and reliable procedure for large-scale fever detection, Dell’Isola et al. [58] proposed to perform a double-step measurement protocol. In the first step, contactless body temperature measurements were provided, setting a temperature threshold of 37.5 °C, considering all the measurement uncertainty due to the real operative measurement conditions. When the first step delivered a temperature value that fell within the uncertainty zone, a second step was needed to provide further contact body temperature measurements. 

The principal performances obtained for the IRI-based mass screening in hospitals and airports during epidemic outbreaks are summarized in Table 1.

## 5. Machine Learning Applications for Respiratory Diseases Assessment

In order to better exploit temporal and spatial features of IRI for infections detection, ML approaches have been proposed. Sun et al. [59] proposed a method for fever assessment based on neural network and fuzzy clustering method during the avian influenza H5N1 pandemic. The method was based on the combined employment of a thermal imager, a microwave radar for the respiration rate assessment, and a finger-tip photo-reflector to measure the heart rate. They obtained a classification of the disease with a sensitivity of 97.1% and a specificity of 81.3%. Dagdanpurev et al. [60] proposed a model for infection screening at various ambient temperatures. Combining a linear regression analysis, to account for environmental thermal condition, with a k-nearest neighbor algorithm, a sensitivity of 91% and NPV of 92% in fever detection were obtained. Ng et al. [61] proposed a method for fever assessment based on parabolic regression, combined with an Artificial Neural Network and ROC curve. The minimum, maximum, average and standard deviation of the temperature of forehead and eyes regions were used as input of the classifier, reaching a sensitivity >90% and a specificity >80% in fever detection. Ng also studied the effectiveness of infrared systems in mass blind screening to detect subjects with elevated body temperature [62]. Linear regression, ROC analysis, and neural networks-based classification were used to analyze the temperature data collected from various sites on the face on both the frontal and side profiles (i.e., forehead; eye region; average cheeks; nose; mouth closed; average temple; side face; ears; and side temple), achieving a sensitivity of 90.7% and specificity of 75.8%. 

Concerning COVID-19 pandemic, Jiang et al. [63,64,65] proposed a method to identify respiratory pathological patterns based on ML. Particularly, face recognition was performed employing Gaussian pyramid box based on RGB data. Breath pattern was extracted from nostrils region (wearing a protection mask, as usual during the pandemic) from 20 s recordings, maximizing the variance of thermal image sequence. A Bidirectional Gate Recurrent Unit with an ATtention mechanism (BiGRU-AT) was used to classify the breathing pattern using as input respiration data. The authors obtained an accuracy of 83.69%, sensitivity of 90.23% and specificity of 84.61% in detecting respiratory diseases patients. Martinez-Jimenez [66] developed a method to discriminate COVID-19 infected individuals with mild respiratory symptoms from negative healthy volunteers. The temperature asymmetry between the lacrimal caruncles and the forehead was significantly higher in COVID-19 patients. Through a random forest analysis, a cut-off value of 0.55 °C was found to discriminate COVID-19 patients from healthy subjects with an accuracy of 82%. 

## 6. Smart Technologies to Limit COVID-19 Diffusion

COVID-19 outbreak urged the development of smart technologies able to detect feverish status and respiratory infections for an early identification of the disease. Particularly, Jiang et al. [64] developed an algorithm able to detect respiratory diseases employing a RGB and thermal camera embedded in a mobile phone, employing 10 s lasting measurements. Al-Humairi et al. [67] conceptually designed an adaptive monitoring system composed of a smart artificial intelligence helmet, with an IRI embedded system. Rane [68] demonstrated the feasibility of using a humanoid robot with infrared sensors for fever detection. The system required a Raspberry Pi controller and an IRI device, and allowed to automatically detect, through ML algorithms, nose tip, chin, and inner canthi. Mohammed et al. [69] proposed a smart helmet with an embedded IRI system that allowed to automatically detect subjects’ face through OpenCV [57] libraries and to support the identification of COVID-19 by means of ML algorithms. Sun et al. [70] implemented a multiple vital-signs-based infection screening system called KAZEKAMO, based on a thermopile. Employing Kohonen’s self-organizing map (SOM) and k-means clustering algorithm, a sensitivity in fever screening of 88% was obtained. Finally, Kumar et al. [71] proposed an ML-based system that collected thermal data of people in real-life scenarios through drones: if the recorded temperature of any person was greater than at least 2 °C than normal body temperature, an alarm beeped and an announcement was made over the loudspeaker fitted on the drone. 

Concerning healthcare settings applications, Tsai et al. [72] developed a contactless wireless sensor, based on self-injection-locked radar and IRI, placed on the ceiling of the ward. The system could automatically detect and record the vital signs of the patient every five seconds, fostering the isolation policy in hospitals, thus preserving the healthcare staff from the infection. Sun et al. [73] employed a CMOS camera-equipped IRI system (TVS-500; NEC/AVIO Infrared Technologies Co. Ltd., Tokyo, Japan) to assess fever in clinical settings, in order to promote the remote sensing of vital signs. Particularly, from IRI videos the breathing and heart rates were estimated. A logistic regression discriminant function predicted the likelihood of infection with sensitivity of 87.5%, specificity of 100%, NPV of 91.7%, and PPV of 100%. 

## 7. Discussion

The present review aimed to investigate the state of the art of the employment of IRI for fever mass screening, focusing on the methods of analysis currently employed for infections identification during epidemics. During the spread of the COVID-19 disease, the employment of IRI technologies was fostered to detect the feverish condition in public buildings, such as airports and hospitals, to limit the diffusion of the infection. Several scientific publications, reported in the present review, dealt with the usage of IRI technology for mass fever screening, revealing its great performances of IRI in this regard. Moreover, in order to make the fever detection more accurate and quicker, algorithms for the automatic identification of the eye’s inner canthi, which are highly indicative of the core temperature, were implemented. It is worth to mention that also other facial regions (e.g., forehead) have been demonstrated to be indicative of the core temperature. Anyway, in ecological conditions, such as large-scale fever screening, the inner canthus is proved to be less sensitive to external confounding, such as the environmental temperature, the employment of make-up and sweating [74,75]. 

However, some COVID-19 patients do not exhibit fever or are asymptomatic, hence the only evaluation of the fever is not exhaustive for the detection of the infection. For this reason, some ML-based approaches have been developed to identify the pathology (e.g., evaluating breathing rate altered pattern). However, few studies investigating COVID-19 patients through IRI are available, hence the findings of the literature are mostly proof of concept of the feasibility of employing this technology for the detection of the disease. 

From the review of the literature, some suggestions can be proposed to effectively employ IRI for the detection of the COVID-19. Firstly, algorithms for the automatic detection of human faces could facilitate the identification of the inner canthi. Moreover, these algorithms could provide the position of other facial landmarks, hence allowing to evaluate not only the feverish state but also the spatial facial temperature distribution, that could be indicative of ongoing infections. Furthermore, it could be worth considering also temporal features from IRI recordings, such as the temperature time course on relevant regions of interest (e.g., the nostrils, indicative of the breathing rate), employing modelling approaches, frequency-domain and non-linear methods of analysis [76,77]. Although a high speed of detection is preferred for mass screening in public contexts, measurements lasting some seconds should be recommended to exploit also temporal features of the thermal recordings. Particularly, in the literature examined, the shorter measurements employed to detect respiratory diseases were 10 s, hence measurements of at least this duration should be performed. However, it is worth to note that the respiratory breath generally ranges between 15–18 breaths per minute in healthy adults, hence shorter measurements could be suitable to detect respiratory pathologies, but further studies are needed to investigate this aspect. 

Modern ML algorithms, such as deep learning, could prevent the issue of the definition of regions of interest on the face, given their ability to automatically extract relevant features, optimizing the classification performances. However, the effectiveness of IRI in detecting COVID-19 should be extensively tested on patients and, particularly, on asymptomatic patients, in order to fully explore the potentiality of IRI to stem the tide of this pandemic. In fact, the capability of IRI to discriminate COVID-19 patients with mild respiratory symptoms relies on the evaluation of the difference of temperature between facial regions of interest [66]. Hence, further studies should test the capability of ML to increase the IRI diagnostic capabilities in discriminating even asymptomatic patients investigating the facial temperature pattern.

## 8. Conclusions

IRI is a non-invasive, contactless and low-cost technology able to measure T_sk_ modulations. Its contactless features and its ability to detect fever in ecological conditions are exploited during pandemics, with fever symptoms, to limit the diffusion of the infection. This review investigated the potentialities of IRI employment during pandemics to contain the contagions. Furthermore, the review focused on the major issues related to the automatic face recognition and ROIs selection for the rapid fever assessment, and the statistical analysis employed to increase the fever diagnostic capability of IRI. The evaluation of the maximum temperature from the eye inner canthus seems to be the most reliable method to assess fever, hence the development of automatic and rapid methods for facial landmarks detection is fundamental. Moreover, ML frameworks could constitute a suitable tool, to exploit both spatial and temporal IRI features, potentially providing optimal classification performances. 

## Figures and Tables

**Table 1:** Performances of IRI-based mass screening in epidemics.

Authors	Pathology	Environment	Sensitivity	Specificity	PPV	NPV
Hewlett et al. [45]	Fever	Hospital	0.70	0.92	0.42	0.97
Chiu et al. [46]	Fever	Hospital	0.75	1.00	-	-
Bardou et al. [47]	Fever	Hospital	0.93	1.00	0.87	1.00
Nishiura and Kamiya [51]	H1N1	Airport	0.51–0.70	0.64–0.82	-	-
Kuan et al. [52]	Dengue fever	Airport	-	-	0.31–0.63	-
Kuan and Chang [53]	Dengue fever	Airport	-	-	0.24	1.00
Sun et al. [56]	SARS	Airport	1.00	0.88	0.33	1.00

## Data Availability

Data sharing not applicable.
